# Robust Coarse-to-Fine Registration Scheme for Mobile Laser Scanner Point Clouds Using Multiscale Eigenvalue Statistic-Based Descriptor †

**DOI:** 10.3390/s21072431

**Published:** 2021-04-01

**Authors:** Yongjian Fu, Zongchun Li, Wenqi Wang, Hua He, Feng Xiong, Yong Deng

**Affiliations:** School of Geospatial Information, PLA Strategic Support Force Information Engineering University, Zhengzhou 450001, China; yongjianfu@aliyun.com (Y.F.); wenqi_xd@sina.com (W.W.); hexiaoshuai_xd@aliyun.com (H.H.); 766007074@cug.edu.cn (F.X.); gc_alliance@aliyun.com (Y.D.)

**Keywords:** MLS point clouds, pairwise registration, weighted covariance matrix, multiscale eigenvalues

## Abstract

To overcome the drawbacks of pairwise registration for mobile laser scanner (MLS) point clouds, such as difficulty in searching the corresponding points and inaccuracy registration matrix, a robust coarse-to-fine registration method is proposed to align different frames of MLS point clouds into a common coordinate system. The method identifies the correct corresponding point pairs from the source and target point clouds, and then calculates the transform matrix. First, the performance of a multiscale eigenvalue statistic-based descriptor with different combinations of parameters is evaluated to identify the optimal combination. Second, based on the geometric distribution of points in the neighborhood of the keypoint, a weighted covariance matrix is constructed, by which the multiscale eigenvalues are calculated as the feature description language. Third, the corresponding points between the source and target point clouds are estimated in the feature space, and the incorrect ones are eliminated via a geometric consistency constraint. Finally, the estimated corresponding point pairs are used for coarse registration. The value of coarse registration is regarded as the initial value for the iterative closest point algorithm. Subsequently, the final fine registration result is obtained. The results of the registration experiments with Autonomous Systems Lab (ASL) Datasets show that the proposed method can accurately align MLS point clouds in different frames and outperform the comparative methods.

## 1. Introduction

Point clouds obtained with modern three-dimensional (3D) sensors, such as mobile laser scanner (MLS), have played an important role in civil and transportation engineering [1,2,3], forest structure monitoring [4,5], and spatial deformation monitoring [6,7]. However, due to errors in the calibration and positioning of sensors, MLS point clouds obtained from different frames or periods suffer deviations, several tens of centimeters and even to meters [8]. This impedes the application of MLS point clouds, such as in change detection and deformation monitoring. Therefore, the point clouds in multiple frames or periods must be registered before using them in the application of deformation monitoring, urban management, and similar processes.

Numerous studies have been carried out on point clouds registration [9,10,11], which can be divided into two groups comprising pairwise and multiview registration, depending on the amount of input point clouds. Most pairwise and multiview point cloud registration methods employ a coarse-to-fine strategy [12]. In particular, coarse registration algorithms can be further divided into four categories [13], hand-crafted feature-based registration methods, deep learning-based registration methods, four-points congruent set (4PCS)-based registration method, and probabilistic registration methods. And the fine registration methods mainly include iterative closest point (ICP) [14,15,16] and normal distribution transform (NDT)-based algorithms [17,18,19].

In the process of hand-crafted feature-based registration, coarse registration algorithms, such as random sample consensus (RANSAC) [20], are first applied to estimate the initial transformation between two adjacent point clouds. Next, fine registration algorithms, such as ICP, are utilized to refine the approximate rotation matrix and translation vector. The core of hand-crafted feature-based registration is the correspondence estimation in coarse registration, which is usually matched by the 3D surface feature descriptor. The traditional descriptors include 3D shape context (3DSC) [21], point feature histogram (PFH) [22], fast point feature histogram (FPFH) [23], signature of histogram of orientations (SHOT) [24], and binary shape context (BSC) [25]. Although these feature description languages are sufficiently descriptive, they also consume a lot of time during generation and matching owing to their higher dimensionality, i.e., 3DSC (1980 dimensions), SHOT (352 dimensions), and PFH (125 dimensions).

Compared with traditional descriptors, deep learning-based methods [26,27] can directly learn deep-level feature representations from a mass of data to achieve appropriate performance in terms of descriptiveness and robustness. This type of method has proven effective for the registration of indoor and small-scale point clouds; however, it is difficult to apply it to the registration of large-scale MLS and terrestrial laser scanner (TLS) point clouds because of the limitations related to the amount of data and complexity [13].

4PCS-based methods [28,29,30,31] achieve registration by repeating the following process to obtain the optimal solution: (1) randomly selecting geometrically consistent point pairs; (2) computing the registration matrix and the root mean square error (RMSE) between two-point clouds. The 4PCS scheme works well for datasets with small overlaps, and it requires no assumptions regarding the initial positions. However, the iterative procedure of matching the correspondences and rejecting the mismatched ones is time-consuming.

Coherent point drift (CPD)-based methods [32,33,34], which represents the probabilistic registration method, consider registration as a probability density estimation problem. They first use the Gaussian mixture models (GMM) centroids to describe the source point cloud, and then fit the GMM to the target point cloud by maximizing the likelihood of the objective function. These methods exhibit generality, accuracy, and robustness to noise and outliers. However, because the registration result depends on the sampling result, the method cannot simultaneously deal with large volume points.

The representative fine registration method is the ICP and the NDT algorithm. This type of method can achieve high-quality and high-precision registration by repeating point matching and transformation calculations. However, both ICP and NDT-based methods require a better initial transformation matrix to avoid congregation of points at a local optimum.

In this study, we defined a 3D surface feature descriptor using multiscale eigenvalue statistic to estimate the corresponding points between the MLS point clouds obtained from different frames. With these estimated correspondences, we performed coarse registration. Then, the result of the coarse registration was used as the initial value for the fine registration, such as ICP, to obtain a better result. The major contributions of this study are summarized as follows:

1. A new 3D local descriptor with fewer dimensions (21 dimensions) was proposed to describe the keypoint under multiscale support radii.
2. The proposed descriptor was further used to identify the corresponding points from the different frames of MLS point clouds.

The remainder of this manuscript is organized as follows: The proposed MEVS descriptor is defined in Section 2; Section 3 describes the coarse-to-fine registration scheme; and Section 4 introduces the experiments. The study is concluded in Section 5.

## 2. Multiscale Eigenvalues Statistic-Based Descriptor

In this section, we defined a novel 3D local feature descriptor, named the multiscale eigenvalue statistic (MEVS), to describe the keypoint using multiple eigenvalues obtained under multiscale support radii. The procedure has three main steps: (1) computing point-density and Euclidean-distance-related weights, (2) constructing the weighted covariance matrix, and (3) constructing the MEVS descriptor.

The keypoint and its neighbors are regarded as $N=\{q_{0},q_{1},q_{2},\cdots q_{m}\}$, where $q_{0}$ represents the keypoint, $q_{j}(j=1,2,\ldots m)$ represents the *j*-th nearest neighbor, and *m* is the number of neighboring points. The covariance matrix constructed for $q_{0}$ using N is denoted by *C*, and the support radius for neighboring points searching is expressed as $r^{\mathrm{scale}}$.

### 2.1. Weight Assignment

First, we estimated the point-density-related weight $w_{j}^{\mathrm{density}}$ by Equation (1), the aim is to describe the surface shape of the point better

(1) $$w_{j}^{\mathrm{density}}=\frac{1}{\#\left\{p_{n}:\left\|p_{n}-q_{j}\right\|<r^{\mathrm{density}}\right\}},$$

where $r^{\mathrm{density}}$ represents the radius for point-density estimation, $p_{n}$ is the neighboring point of $q_{j}$, $\left\|p_{n}-q_{j}\right\|$ represents the 3D Euclidean distance between $q_{j}$ and $p_{n}$, and $\#\left\{p_{n}:\left\|p_{n}-q_{j}\right\|<r^{\mathrm{density}}\right\}$ represents the number of points within $r^{\mathrm{density}}$, if it is equal to zero, then we just set $w_{j}^{\mathrm{density}}$=1. By varying $r^{\mathrm{density}}$ from 0.1$r^{\mathrm{scale}}$ to $r^{\mathrm{scale}}$ in intervals of 0.1$r^{\mathrm{scale}}$, we found that the MEVS descriptor performed best at an $r^{\mathrm{density}}$ of 0.5$r^{\mathrm{scale}}$ by testing. Thus, we set $r^{\mathrm{density}}$ = 0.5$r^{\mathrm{scale}}$. The weight $w_{j}^{\mathrm{density}}$ was used to compensate for varying point density; thus, the points in regions with low point density contribute more than those in the dense regions.

Next, the Euclidean-distance-related weight $w_{j}^{\mathrm{distance}}$ was calculated by Equation (2)

(2) $$w_{j}^{\mathrm{distance}}=\frac{r^{\mathrm{scale}}-\left\|q_{0}-q_{j}\right\|}{r^{\mathrm{scale}}}.$$

The weight $w_{j}^{\mathrm{distance}}$ is expected to improve the robustness of the MEVS descriptor, for the distant points contribute less to the overall covariance matrix.

### 2.2. Weighted Covariance Matrix

By using the point set $N=\{q_{0},q_{1},q_{2},\cdots q_{m}\}$ and the corresponding weights for $q_{j}$, we calculated the weighted covariance matrix *C* as follows

(3) $$C=\frac{1}{\sum_{q_{j\in N}}w_{j}^{\mathrm{density}}\cdot w_{j}^{\mathrm{distance}}}\sum_{q_{j\in N}}w_{j}^{\mathrm{density}}\cdot w_{j}^{\mathrm{distance}}\left(q_{0}-q_{j}\right){\left(q_{0}-q_{j}\right)}^{T}.$$

### 2.3. MEVS Descriptor

Eigenvalues $\left\{\lambda_{1},\lambda_{2},\lambda_{3}\right\}$ in the decreasing order of magnitude were obtained by decomposing the weighted covariance matrix *C* and further normalized by Equation (4)

(4) $$\lambda^{C}{}_{i}=\lambda_{i}/\sum_{i=1}^{3}\lambda_{i}.$$

It is assumed that the initial support radius is *R*, by varying R’=R+j·mr(j=1,2,⋯k), where *k* represents the multiscale dimensions and mr represents the mean resolution of the point cloud (in this paper, its unit is meter), we calculated a set of weighted covariance matrices $C_{j}$ and consequently obtain various combinations of eigenvalues. Through normalization, we set $EV_{j}=\left(\lambda_{1}^{C_{j}},\lambda_{2}^{C_{j}},\lambda_{3}^{C_{j}}\right)$. Then, the MEVS descriptor was defined by Equation (5)

(5) $$MEVS=\left\{EV_{1},EV_{2},\cdots EV_{k}\right\}.$$

The MEVS descriptor is expected to be highly viewpoint invariant, because each covariance matrix $C_{j}$ is real and symmetric, so that its eigenvalues do not change when the point set $N_{j}$ is rotated, and each covariance matrix $C_{j}$ remains the same when the point set $N_{j}$ is translated. Because $C_{j}$ is computed from data points, which change and also the density of which changes when the same area is scanned from different viewpoints, the MEVS descriptor is not perfectly viewpoint invariant, but the density-based weighting can improve the viewpoint invariance.

### 2.4. MEVS Generation Parameters

The MEVS feature descriptor had two important parameters: (1) initial support radius *R* and (2) multiscale dimensions *k*. The performance of the MEVS descriptor under different settings of the two parameters was tested on the tuning datasets using a precision versus recall curve (PR curve) [35].

#### 2.4.1. PR Curve Generation

Given a model point cloud, a scene point cloud, and the ground-truth transformation *T* between them, the PR curve was calculated as follows:

1. A number of keypoints were detected from both the model and scene point clouds using the keypoint detector.
2. The proposed MEVS feature descriptor for each keypoint was computed using the proposed method.
3. The nearest neighbor distance ratio (NNDR) technique was used to match the feature descriptors.

Specifically, the nearest and second nearest neighbors for each MEVS descriptor $\mathrm{MEVS}\left(p_{i}^{\mathrm{model}}\right)$ in the model point cloud were selected from the scene point cloud, which are denoted by $\mathrm{MEVS}\left(p_{j}^{\mathrm{scene}}\right)$ and $\mathrm{MEVS}\left(p_{j’}^{\mathrm{scene}}\right)$, respectively. Then, the ratio between the two distances was calculated as $\left\|\mathrm{MEVS}\left(p_{i}^{\mathrm{model}}\right)-\mathrm{MEVS}\left(p_{j}^{\mathrm{scene}}\right)\right\|/\left\|\mathrm{MEVS}\left(p_{i}^{\mathrm{model}}\right)-\mathrm{MEVS}\left(p_{j’}^{\mathrm{scene}}\right)\right\|$. If the distance ratio was less than a threshold τ, the two feature descriptors $\mathrm{MEVS}\left(p_{i}^{\mathrm{model}}\right)$ and $\mathrm{MEVS}\left(p_{j}^{\mathrm{scene}}\right)$ were considered an estimated match, and their corresponding points $p_{i}^{\mathrm{model}}$ and $p_{j}^{\mathrm{scene}}$ were considered a corresponding point pair. Furthermore, the correspondence was assumed a correct match, if the distance $\left\|p_{i}^{\mathrm{model}}-T\cdot p_{j}^{\mathrm{scene}}\right\|$ was less than a predefined threshold (i.e., half of the initial support radius of keypoint $p_{i}^{\mathrm{model}}$ in this study). Otherwise, it was assigned a false match. Finally, the precision of match assignment was calculated as the number of correct matches with respect to the total number of estimated matches, as in Equation (6)

(6) $$Precision=\frac{\mathrm{The}\;\mathrm{number}\;\mathrm{of}\;\mathrm{correct}\;\mathrm{matches}\;}{\mathrm{The}\;\mathrm{number}\;\mathrm{of}\;\mathrm{estimated}\;\mathrm{mathces}}.$$

Recall was calculated as the number of correct matches with respect to the number of ground-truth corresponding points between the given scene and model point cloud, as in Equation (7)

(7) $$Recall=\frac{\mathrm{The}\;\mathrm{number}\;\mathrm{of}\;\mathrm{correct}\;\mathrm{matches}\;}{\mathrm{The}\;\mathrm{number}\;\mathrm{of}\;\mathrm{ground}-\mathrm{truth}\;\mathrm{corresponding}\;\mathrm{points}}.$$

The PR curve can be obtained by varying the threshold τ from 0 to 1. Ideally, the PR curve should be located on the top-right corner of the precision-recall coordinate system, and the larger the area under the PR curve, the more descriptive the descriptor. We tested the performance of the descriptor by examining the different combinations of the two main parameters. By calculating the area under the PR curve, as shown in Table 1, we found that the optimal value of the initial support radius *R* was 12 mr, and the optimal value of the multiscale dimension *k* was 7.

#### 2.4.2. Initial Support Radius

**Figure 1:**
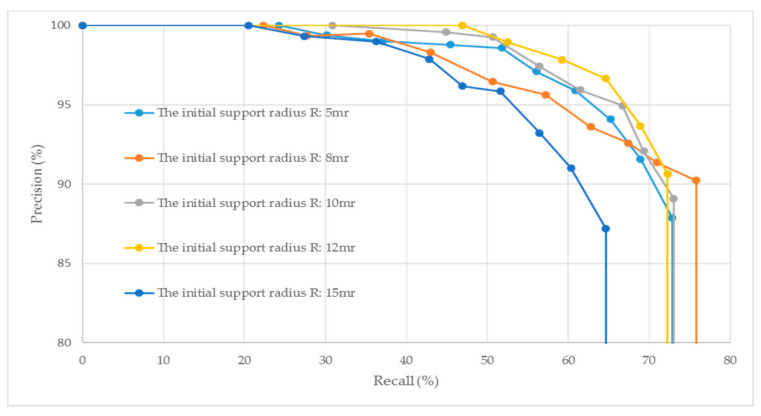
PR curves generated under different initial support radii *R* with the multiscale dimension *k* = 7.

The initial support radius is an important parameter for the generation of the MEVS feature descriptor because it determines both the descriptiveness and robustness of the descriptor. We tested the performance of the MEVS descriptor with respect to varying initial support radii, with the multiscale dimension set at *k* = 7. Figure 1 illustrates the generated PR curves under the initial support radius *R* ranging from 5 mr to 15 mr.

As shown in Figure 1, the MEVS local feature descriptor generated with a small initial support radius *R* (e.g., *R* = 5 mr) cannot eliminate the effect of noise. Hence, the descriptor generated with a small *R* is less robust. Moreover, a small initial support radius allowed the generator to consider less local information, leading to its low descriptiveness. A large initial support radius *R* (e.g., *R* = 15 mr) is more sensitive to occlusion, thereby reducing the descriptiveness of the generated descriptor. We found that the MEVS generated with *R* = 12 mr can optimize local surface shape information and robustness. Therefore, in practice, we used *R* = 12 mr as the initial support radius.

#### 2.4.3. Multiscale Dimension

**Figure 2:**
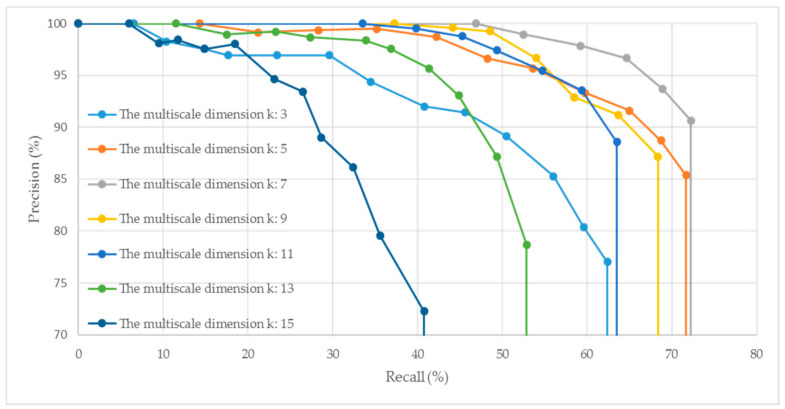
PR curves generated under different multiscale dimension *k* with an initial support radius of *R* = 12 mr.

The multiscale dimension, which determines the maximum search region and dimension of the MEVS descriptor, is another important parameter determining the robustness and descriptiveness of the descriptor. We tested the performance of the descriptor by varying the values of the multiscale dimension, with the initial support radius set to *R* = 12 mr. Figure 2 illustrates the PR curves generated under different multiscale dimensions *k*.

As shown in Figure 2, the descriptiveness and robustness of the generated MEVS feature descriptor first increase with an increase in the multiscale dimension *k* (e.g., from 3 to 7), and then weaken with the further increase of *k* (e.g., from 7 to 15). This phenomenon occurs because *k* determines the maximum range of the neighborhood search, which directly affects the descriptiveness and robustness of the local descriptor. To improve the descriptiveness, a smaller support radius should be used. Meanwhile, to enhance the robustness of the MEVS descriptor, the support radius should be increased appropriately, but not so much that it increases the sensitivity of the descriptor to occlusion and confusion. Therefore, we used *k* = 7 as the multiscale dimension.

## 3. Coarse-to-Fine Pairwise Registration

**Figure 3:**
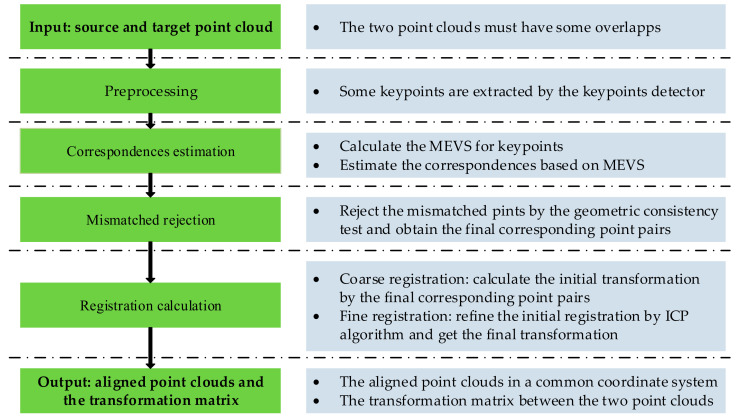
Flowchart of the proposed coarse-to-fine registration algorithm.

The proposed coarse-to-fine pairwise registration scheme primarily included the following steps: (1) correspondences estimation, (2) mismatches rejection, and (3) registration calculation. Figure 3 demonstrates the specific process of the proposed algorithm.

### 3.1. Correspondences Estimation

To improve the efficiency, some keypoints were extracted from the original point cloud using a keypoint detector, such as 2.5D SIFT [36,37], 3D Harris [38], NARF [39], and intrinsic shape signatures (ISS) [40]. As shown in [41] that the ISS detector performed the best, we adopted the ISS algorithm to detect the keypoints.

A bidirectional matching strategy [42] was applied in which the keypoints extracted from the source and target point clouds were regarded as $KP_{S}=\left\{p_{1}^{s},p_{2}^{s},\cdots p_{Ts}^{s}\right\}$ and $KP_{T}=\left\{p_{1}^{t},p_{2}^{t},\cdots p_{Tt}^{t}\right\}$, respectively. The corresponding MEVS was $M_{S}=\left\{m_{1}^{s},m_{2}^{s},\cdots m_{Ts}^{s}\right\}$ and $M_{T}=\left\{m_{1}^{t},m_{2}^{t},\cdots m_{Tt}^{t}\right\}$. For each $m_{k}^{s}$ from $M_{S}$, if there exists an element $m_{h}^{t}$ in $M_{T}$ that satisfies the constraint as in Equation (8), then $m_{k}^{s}$ and $m_{h}^{t}$ together were considered matched descriptors. In other words, only when $m_{h}^{t}$ is the nearest descriptor to $m_{k}^{s}$ in $M_{T}$, and $m_{k}^{s}$ to $m_{h}^{t}$ in $M_{S}$, then $m_{k}^{s}$ and $m_{h}^{t}$ were the corresponding descriptors, and the corresponding points were regarded the matched point pair

(8) $$\left\{\begin{array}{c} h=\arg\min\left\|m_{k}^{s}-m_{n}^{t}\right\|(n=1,2,\cdots N_{t})\\ k=\arg\min\left\|m_{h}^{t}-m_{n}^{s}\right\|(n=1,2,\cdots N_{s}) \end{array}\right.,$$

where the norm is the 21D Euclidean distance.

All the matched point pairs calculated by the above procedure were set as $FC=\left\{c_{1},\cdots c_{m},\cdots c_{M_{FC}}\right\}$, where $c_{m}=\left\{m_{m}^{s},m_{m}^{t}\right\}$ represents the *m*-th corresponding point pair, and $M_{FC}$ is the length of FC.

### 3.2. Mismatches Rejection

In principle, after obtaining *FC*, the transformation from the source to target point clouds can be directly calculated; however, there were some mismatches in *FC*, which lowed the accuracy of registration, even leading to incorrect result. Therefore, we introduced geomatic consistency constraint [43] to remove the mismatches from *FC*. If two correspondences in *FC*, named $c_{m}=\left\{m_{m}^{s},m_{m}^{t}\right\}$ and $c_{n}=\left\{m_{n}^{s},m_{n}^{t}\right\}$, satisfy Equation (9), then they are regarded as the right corresponding point pairs

(9) $$abs\left(\left\|p_{m}^{s}-p_{n}^{s}\right\|-\left\|p_{m}^{t}-p_{n}^{t}\right\|\right)<\varepsilon ,$$

where $p_{m}^{s}$, $p_{m}^{t}$, $p_{n}^{s}$, and $p_{n}^{t}$ are the points corresponding to the descriptors $m_{m}^{s}$, $m_{m}^{t}$, $m_{n}^{s}$, and $m_{n}^{t}$, respectively. ε is a threshold, which, in this study, was set as 5 mr. $abs\left(\cdot \right)$ represents the absolute value and ‖ ‖ is the 3D Euclidean distance between the two points.

For each $c_{m}=\left\{m_{m}^{s},m_{m}^{t}\right\}$ in *FC*, we traverse *FC* to determine the correspondences that satisfy Equation (9), and the results combined with $c_{m}=\left\{m_{m}^{s},m_{m}^{t}\right\}$ are regarded as a group. Finally, we obtained $M_{FC}$ groups. The more elements in a group, the more right corresponding point pairs it may contain. Thus, we chose the largest group as the final matched point pairs $GC=\left\{c_{1},\cdots c_{m},\cdots c_{M_{GC}}\right\}$.

### 3.3. Registration Calculation

The registration adopts a coarse-to-fine strategy. First, coarse registration is performed using the point set *GC*. Second, the transformation obtained by coarse registration is further refined by the ICP algorithm. The coarse registration uses the corresponding points to estimate roughly yet quickly the transformation between the source and target point clouds. By iteratively random sampling *GC*, we can get a series $\delta_{m}$ according to Equation (10), when $\delta_{m}$ is less than a threshold, the coarse registration matrix $T_{s,t}^{C}$ is obtained

(10) $$\delta_{m}=\sum_{j=1}^{L_{m}}{\left\|T_{s,t}^{C}\cdot p_{j}^{s}-p_{j}^{t}\right\|}^{2},$$

where $p_{j}^{s}$ and $p_{j}^{t}$ are the *j*-th corresponding points in *GC*, $T_{s,t}^{C}$ is the coarse registration matrix, $L_{m}$ is the length of a sample of *GC*, and $\delta_{m}$ is the sum of squares of the Euclidean distance between the corresponding points after transformed to the same coordinate system.

The goal here is not pairwise coarse registration itself but to provide a robust, reliable initial transformation for fine registration [44]. Therefore, $T_{s,t}^{C}$ was further refined by the ICP algorithm to obtain a better registration matrix $T_{s,t}^{F}$, which is regarded as the final transformation between source and target point clouds.

## 4. Experiments and Analysis

In this section, we test the performance of the proposed coarse-to-fine registration method by evaluating the effectiveness of the keypoint matching using MEVS for registration. Accordingly, we design Method1, in which we use the RANSAC algorithm to directly address the extracted keypoints to behave the coarse registration with the unit matrix as the initial value. This value initialized the ICP algorithm to address the source and target point clouds and obtain the fine registration matrix.

Test the advantages and disadvantages of keypoint matching using MEVS for registration, we design Method2 and Method3. In Method2, we used the sample consensus initial alignment (SAC-IA) [23] algorithm with the FPFH descriptor to address keypoints to obtain the coarse registration; In Method3, we used the corresponding points from the source and target point clouds identified by the SHOT descriptors to obtain the coarse registration with RANSAC algorithm; And the coarse registration was further refined using the ICP algorithm. As suggested by [12], we set the parameter of the support radius for FPFH and SHOT as 15 mr.

All the experiments were implemented on a ThinkPad X1 Extreme laptop with an Intel Core i7-9750h CUP @ 2.6 GHz clock speed and 16 GB RAM.

### 4.1. Data Description

**Figure 4:**
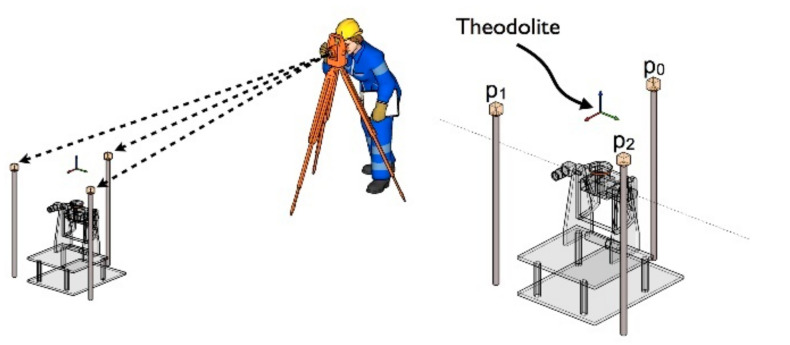
The illustration of obtaining the ground-truth. (https://projects.asl.ethz.ch/datasets/doku.php?id=hardware:tiltinglaser, accessed on 8 March 2021).

**Figure 5:**
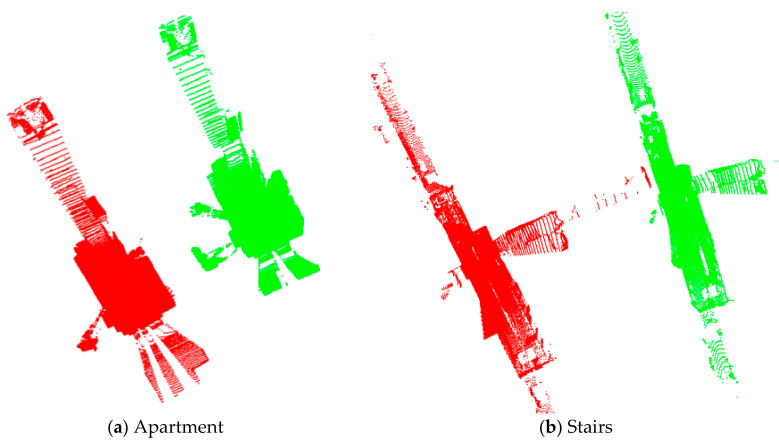

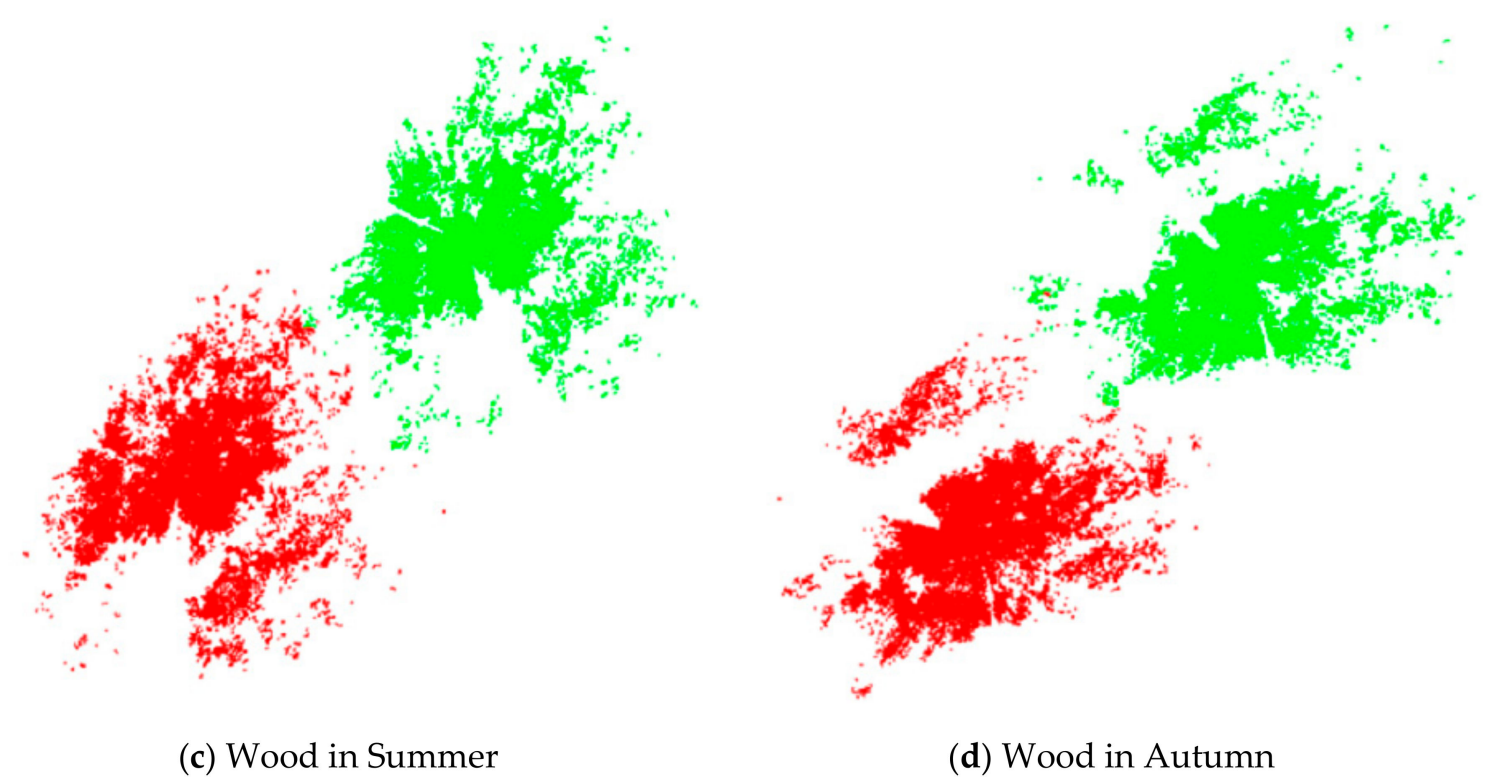
Original point clouds. (**a**–**d**) is the dataset of Apartment, Stairs, Wood in Summer, and Wood in Autumn, respectively. The red and green points represent the source and target point clouds, respectively.

**Figure 6:**
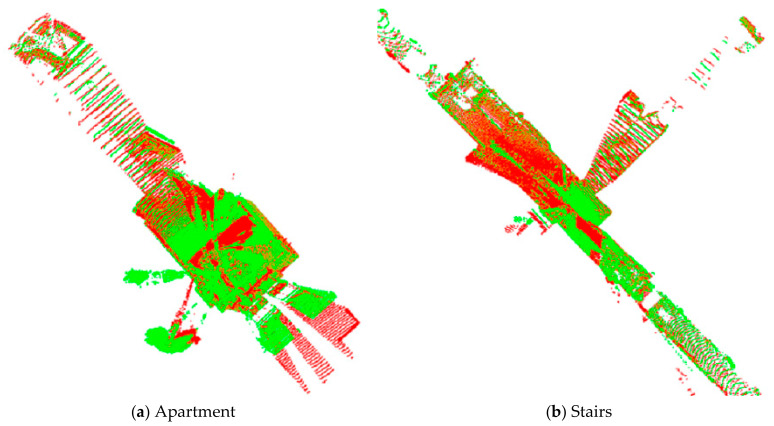

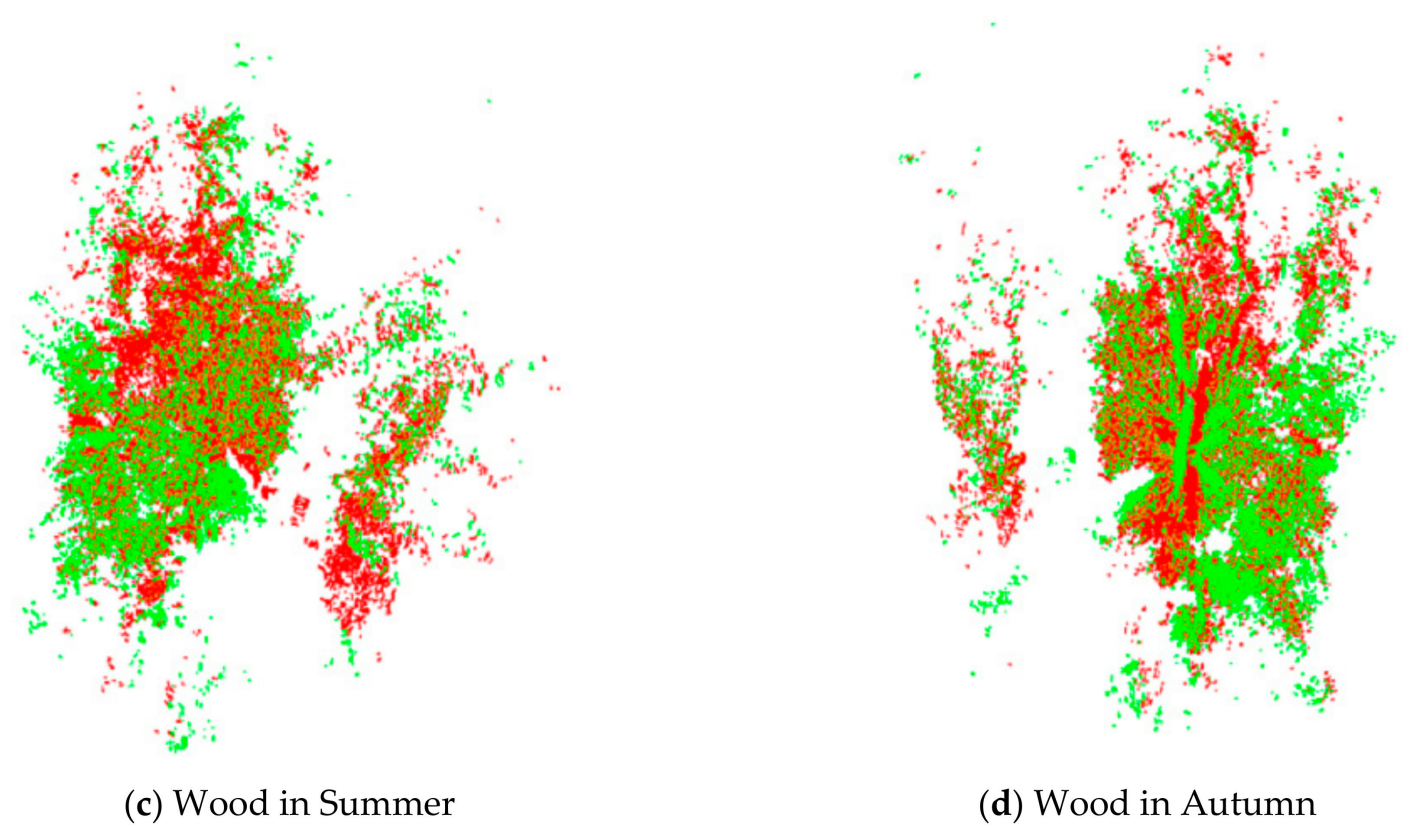
Results of point cloud registration by the ground-truth. (**a**–**d**) is the registration results of Apartment, Stairs, Wood in Summer, and Wood in Autumn, respectively. The red and green points represent the source and target point clouds, respectively.

Four datasets from the ASL Datasets Repository [45] were used to test and evaluate the performance of the proposed and existing methods. These data sets were collected to verify registration algorithms for point clouds obtained in specific environments and conditions. The different point clouds are characterized by diverse environments and geometric primitives. And the data were collected used a custom-made rotating scanner (Hokuyo UTM-30LX), and its precision is about ± 3 cm, and the ground-truth was obtained by TS15, a theodolite from Leica Geosystems, as shown in Figure 4. Figure 5 shows the original four datasets used in this study. Figure 6 summarizes the registration results by the ground-truth (the ground-truth are listed in Table 2), in which the different color represents the point clouds in different frames. Table 3 details the information about the four datasets.

### 4.2. Evaluation Criteria

The criteria followed to evaluate for the performance of the proposed and comparative methods are the measurements of rotation error, translation error, and registration efficiency, which are commonly used for the evaluation of point cloud registration [46,47].

Given a source point cloud $P_{s}$, the transformation $T_{s,t}$ from $P_{s}$ to the target point cloud $P_{t}$ can be calculated using the proposed and comparative methods. The residual transformation is $\Delta T_{s,t}$, defined as

(11) $$\Delta T_{s,t}=T_{s,t}{\left(T_{s,t}^{G}\right)}^{-1}=\left[\begin{array}{cc} \Delta R_{s,t} & \Delta t_{s,t}\\ 0 & 1 \end{array}\right],$$

where $T_{s,t}$ is the estimated transformation from $P_{s}$ to $P_{t}$, and $T_{s,t}^{G}$ is the corresponding ground-truth transformation.

Then, the rotation error $e_{s,t}^{r}$ and translation error $e_{s,t}^{t}$ form $P_{s}$ to $P_{t}$ were calculated based on their corresponding rotation component $\Delta R_{s,t}$ and translation component $\Delta t_{s,t}$, as follows

(12) $$\left\{\begin{array}{l} e_{s,t}^{r}=\arccos\left(\frac{tr(\Delta R_{s,t})-1}{2}\right)\\ e_{s,t}^{t}=\left\|\Delta t_{s,t}\right\| \end{array}\right.,$$

where $tr(\Delta R_{s,t})$ denotes the trace of $\Delta R_{s,t}$, and the rotation error $e_{s,t}^{r}$ corresponds to the angle of rotation in the axis-angle representation.

### 4.3. Results and Discussion

#### 4.3.1. Keypoints Processing

**Figure 7:**
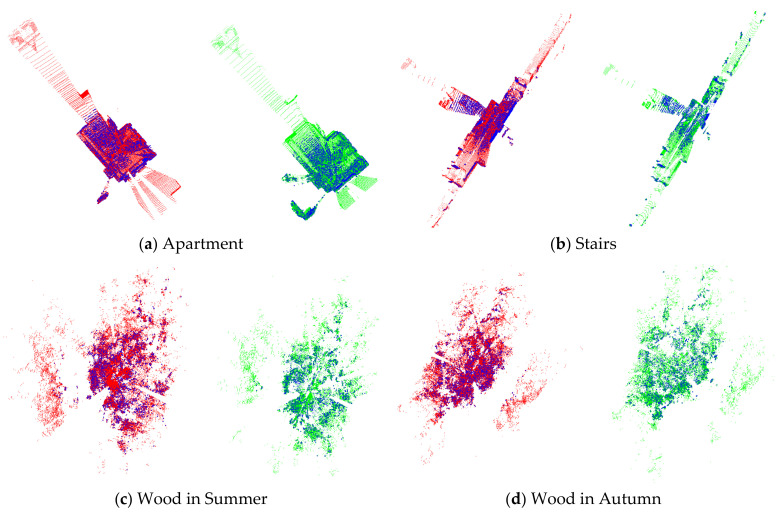
Results of keypoints extraction. The blue points represent the keypoints extracted by the intrinsic shape signatures (ISS) algorithm. The red and green points represent the source and target point clouds, respectively. (**a**–**d**) present the datasets of Apartment, Stairs, Wood in Summer, and Wood in Autumn, respectively.

**Figure 8:**
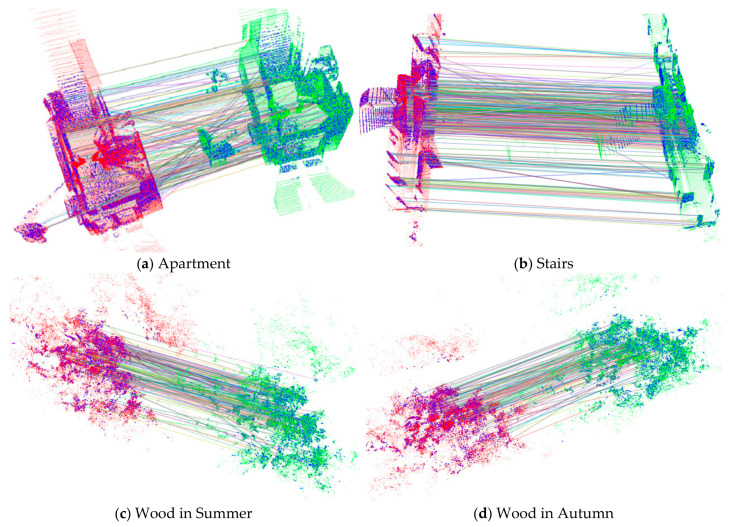
Results of keypoints matching by multiscale eigenvalue statistic (MEVS). The blue points represent the keypoints. The red and green points represent the source and target point clouds, respectively. The line means the corresponding. (**a**–**d**) present the matching results of Apartment, Stairs, Wood in Summer, and Wood in Autumn, respectively.

To improve the efficiency of registration, some keypoints were first extracted from the source and target point clouds using the ISS [40] algorithm. Figure 7 illustrates the results of the keypoint extraction. The matching correspondences estimated by the method proposed in Section 3 are shown in Figure 8. Further details on keypoint processing are listed in Table 4.

The comprehensive analyses of Figure 7 and Figure 8 and Table 4 reveal that (1) the ISS algorithm can extract some significant points from the original point cloud, and (2) MEVS can determine accurate correspondences from the keypoints of the source and target point clouds, and provide high-quality corresponding points for the subsequent rough registration. However, the method still has the following shortcomings: (1) As shown in Figure 7, the spatial distribution of keypoints extracted by the ISS algorithm is not appropriate. For example, the keypoints of the Apartment dataset are concentrated in a relatively dense house, whereas the corridor contains hardly any points; (2) As shown in Figure 8, a few mismatches remain; and (3) As listed in Table 4, the number of final matched keypoints is relatively small, accounting for only 30–40% of the total keypoints. These limitations should be addressed in future studies.

#### 4.3.2. Coarse-to-Fine Registration

**Figure 9:**
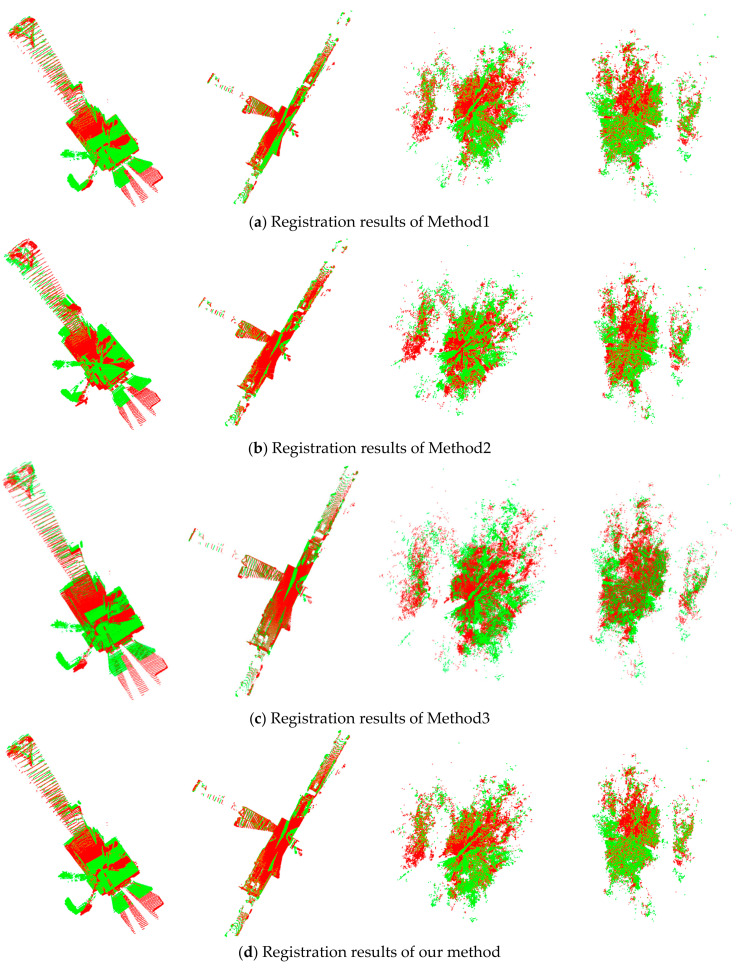
Results of registration. (**a**–**d**) are the registration results of Method1, Method2, Method3, and our method, respectively. From left to right: registration results of Apartment, Stairs, Wood in Summer, and Wood in Autumn, respectively. The red and green points represent the source and target point clouds, respectively.

Figure 9 illustrates the results of registration for the four ASL datasets using the proposed and comparative methods.

As shown by the qualitative testing results in Figure 9, the proposed algorithm can obtain better registration results for the four challenging public datasets, which indicates that it is highly feasible for the registration of datasets obtained from different scenes.

Table 5 lists the rotation and translation errors of coarse and fine registration by different methods.

The following conclusions can be drawn from the analysis of Table 5: (1) The proposed method can achieve suitable registration results between the MLS point clouds in two frames regardless of the type of data [indoor data (Apartment), outdoor data (Wood in Summer and Wood in Autumn), or mixed data (Stairs)]. The rotation and translation have accuracies of more than 0.04 rad and 0.08 m, respectively. (2) The proposed algorithm has a smaller registration error than Method1, indicating that MEVS can effectively describe the local neighborhood information of keypoints. Moreover, it can accurately match keypoints extracted from the source and target point clouds with one another. (3) For the Wood in Autumn dataset, the final registration of the proposed method is slightly inferior to that of Method2 in terms of translation error, but better in terms of the rotation error, so the two methods perform similarly with this dataset. For the Apartment dataset, the final registration of the proposed method is slightly inferior to that of Method3 in terms of translation error, but better in terms of the rotation error, so they perform similarly with Apartment. For the other datasets, the proposed method registers better than Method2 and Method3, which indicates that the MEVS matches keypoints better than FPFH and SHOT. In other words, MEVS is more descriptive and makes less mismatches than FPFH and SHOT, the reason is that it uses a series of radii for the ball nearest neighbor searching, rather than just one radius, to calculate the descriptor, so it can distinguish the correspondences much better. (4) The proposed algorithm can accurately register the data of Wood in Summer and Wood in Autumn, indicating that it is not affected by the change of season. (5) With a rotation error of 0.0316 rad and a translation error of 0.078 m, the proposed method performed the worst with the Apartment dataset, which may have been caused by the dynamic changes during the scanning of the Apartment dataset. With a rotation error of 0.0090 rad and a translation error of 0.023 m, this method performed the best with the Stairs dataset, the reason may be the good spatial distribution of the keypoints.

All of the four methods use ICP for refinement, so it is necessary to consider the efficiency of registration, which is measured in terms of time consumption. Table 6 lists the time consumed by the four methods.

Table 6 reveals that the proposed method required the least number of iterations and computation time, which indicates that it can yield highly reliable coarse registration results as input for ICP or other iterative fine-registration algorithms.

## 5. Conclusions

Thus far, it has been difficult to determine accurate corresponding points and achieve reliable registration for different frames of MLS point cloud. To solve this problem, a new 3D local descriptor with fewer dimensions (21 dimensions) was proposed to describe the keypoint under multiscale support radii, which was further used to estimate the correspondences from the MLS point clouds in different frames. With these correspondences, we proposed a coarse-to-fine registration scheme for the MLS point cloud from the pairwise frames. First, coarse registration was conducted using the RANSAC algorithm with the correspondences. Next, the initial value calculated in coarse registration was refined by the ICP algorithm to obtain an optimal one. The proposed coarse-to-fine registration scheme achieved globally optimal registration for four experimental datasets, with maximum rotation and translation errors of 0.0316 rad and 0.078 m, respectively, and minimum rotation and translation errors of 0.009 rad and 0.023 m, respectively. Besides, it has good efficiency, coarsely aligning two-point clouds with 230,000 points (average) in each within 30 s, and refining them within 6 min. However, the ratio of keypoint matching with this method is slightly low, which should be the focus of future research.

## Figures and Tables

**Table 1:** The area under the precision versus recall (PR) curve generated by different combinations of *R* and *k*.

	*R* = 5 mr	*R* = 8 mr	*R* = 10 mr	*R* = 12 mr	*R* = 15 mr
*k* = 3	0.516	0.587	0.583	0.583	0.560
*k* = 5	0.632	0.726	0.696	0.758	0.685
*k* = 7	0.714	0.738	0.714	0.778	0.630
*k* = 9	0.736	0.750	0.670	0.762	0.599
*k* = 11	0.776	0.723	0.625	0.701	0.449
*k* = 13	0.765	0.691	0.512	0.692	0.288
*k* = 15	0.756	0.660	0.377	0.637	0.156

**Table 2:** The ground-truth of the datasets used in this study.

Ground-Truth	Apartment			Stairs			Wood in Summer			Wood in Autumn		
Rotation matrix	0.9931	−0.1164	0.0058	0.9883	0.1524	0.0043	0.9843	−0.1727	−0.0361	0.9895	−0.1438	−0.1438
	0.1164	0.9931	0.0034	−0.1522	0.9878	−0.0301	0.1726	0.9849	−0.0035	0.1440	0.9891	−0.0280
	−0.0062	−0.0027	0.9999	−0.0088	0.0291	0.9995	0.0362	−0.0027	0.9993	−0.0049	0.0290	0.9995
Translation vector	0.6148	−0.0142	0.0085	0.4232	−0.0508	0.0670	0.6057	0.0407	0.0269	0.4946	0.0496	0.0150

**Table 3:** Details of the four datasets.

Datasets	Frames	Mean Point Number	Scene	Spatial Scale (m)	Dynamics
Apartment	45	365,000	Indoors	17 × 10 × 3	Furniture moved between scans
Stairs	31	191,000	Mixed	21 × 111 × 27	Non
Wood in Summer	37	182,000	Outdoors	30 × 53 × 20	Seasonal changes
Wood in Autumn	32	178,000	Outdoors	36 × 60 × 22	Seasonal changes

**Table 4:** Details of keypoints processing.

Datasets	Original Points’ Number	Keypoints’ Number	Matching Keypoints’ Number
Apartment	370,260/370,277	9140/10,436	3102
Stairs	185,850/181,077	6829/7067	2280
Wood in Summer	192,802/183,179	4556/4721	1746
Wood in Autumn	190,499/189,441	4811/5130	1914

**Table 5:** Quantitative evaluation of the registration accuracy.

Datasets	Methods	Coarse Registration		Fine Registration	
		Rotation Error (rad)	Translation Error (m)	Rotation Error (rad)	Translation Error (m)
Apartment	Method1	0.0789	0.336	0.0530	0.165
	Method2	0.8244	1.094	0.2377	0.601
	Method3	0.2336	0.365	0.0399	0.071
	Our method	0.0483	0.145	0.0316	0.078
Stairs	Method1	0.0503	0.139	0.0491	0.139
	Method2	0.2246	0.726	0.0612	0.150
	Method3	0.0602	0.122	0.0214	0.149
	Our method	0.0124	0.084	0.0090	0.023
Wood in Summer	Method1	0.0592	0.330	0.0278	0.092
	Method2	0.2476	1.225	0.0957	0.640
	Method3	0.2559	0.296	0.0238	0.109
	Our method	0.0682	0.132	0.0220	0.039
Wood in Autumn	Method1	0.0634	0.304	0.0503	0.240
	Method2	0.1861	0.545	0.0280	0.050
	Method3	0.3118	0.505	0.0511	0.209
	Our method	0.0503	0.240	0.0125	0.078

**Table 6:** Time consumption of the proposed and comparative methods.

Datasets	Methods	Coarse Registration Time (min)	Fine Registration		Total Time (min)
			Time (min)	Iterations	
Apartment	Method1	0.10	17.71	17	17.81
	Method2	6.51	35.05	31	41.56
	Method3	0.12	29.68	26	29.80
	Our method	0.26	15.25	15	15.21
Stairs	Method1	0.05	6.39	14	6.44
	Method2	4.85	7.62	17	12.47
	Method3	0.06	3.51	10	3.57
	Our method	0.95	2.04	5	2.99
Wood in Summer	Method1	0.04	4.21	14	4.25
	Method2	2.88	9.95	33	12.83
	Method3	0.06	9.92	33	9.98
	Our method	0.08	3.72	13	3.80
Wood in Autumn	Method1	0.04	5.02	15	5.06
	Method2	2.10	16.04	48	18.14
	Method3	0.05	8.49	24	8.54
	Our method	0.10	3.73	11	3.83

## Data Availability

Not applicable.
